# U1 snRNA interactions with deep intronic sequences regulate splicing of multiple exons of spinal muscular atrophy genes

**DOI:** 10.3389/fnins.2024.1412893

**Published:** 2024-07-12

**Authors:** Eric W. Ottesen, Natalia N. Singh, Joonbae Seo, Ravindra N. Singh

**Affiliations:** Department of Biomedical Sciences, Iowa State University, Ames, IA, United States

**Keywords:** pre-mRNA splicing, U1 snRNP, U1 snRNA, spinal muscular atrophy (SMA), survival motor neuron (SMN), super minigene

## Abstract

**Introduction:**

The U1 small nuclear RNA (snRNA) forms ribonucleoprotein particles (RNPs) such as U1 snRNP and U1-TAF15 snRNP. U1 snRNP is one of the most studied RNPs due to its critical role in pre-mRNA splicing in defining the 5′ splice site (5′ss) of every exon through direct interactions with sequences at exon/intron junctions. Recent reports support the role of U1 snRNP in all steps of transcription, namely initiation, elongation, and termination. Functions of U1-TAF15 snRNP are less understood, though it associates with the transcription machinery and may modulate pre-mRNA splicing by interacting with the 5′ss and/or 5′ss-like sequences within the pre-mRNA. An anti-U1 antisense oligonucleotide (ASO) that sequesters the 5′ end of U1 snRNA inhibits the functions of U1 snRNP, including transcription and splicing. However, it is not known if the inhibition of U1 snRNP influences post-transcriptional regulation of pre-mRNA splicing through deep intronic sequences.

**Methods:**

We examined the effect of an anti-U1 ASO that sequesters the 5′ end of U1 snRNA on transcription and splicing of all internal exons of the spinal muscular atrophy (SMA) genes, *SMN1* and *SMN2*. Our study was enabled by the employment of a multi-exon-skipping detection assay (MESDA) that discriminates against prematurely terminated transcripts. We employed an SMN2 super minigene to determine if anti-U1 ASO differently affects splicing in the context of truncated introns.

**Results:**

We observed substantial skipping of multiple internal exons of *SMN1* and *SMN2* triggered by anti-U1 treatment. Suggesting a role for U1 snRNP in interacting with deep intronic sequences, early exons of the *SMN2* super minigene with truncated introns were resistant to anti-U1 induced skipping. Consistently, overexpression of engineered U1 snRNAs targeting the 5′ss of early *SMN1* and *SMN2* exons did not prevent exon skipping caused by anti-U1 treatment.

**Discussion:**

Our results uncover a unique role of the U1 snRNA-associated RNPs in splicing regulation executed through deep intronic sequences. Findings are significant for developing novel therapies for SMA based on deep intronic targets.

## 1 Introduction

Alternative splicing is an essential process by which a single gene generates multiple unique transcripts in a cell-specific manner by differential usage of splice sites (Baralle and Giudice, 2017). Alternative splicing of a given exon is subject to dynamic combinatorial control by numerous proteins and cis-elements, including RNA structure (Singh and Singh, 2018; Baralle et al., 2019; Shenasa and Hertel, 2019; Singh et al., 2022). Overexpression of splicing factors and/or mutations of cis-elements are associated with pathogenic conditions, including neurodegenerative diseases and cancer (Tazi et al., 2009; Lee and Rio, 2015; Schilling et al., 2019; Wei et al., 2024). U1 snRNP, an RNA-protein particle (RNP) comprised of U1 snRNA, seven Sm proteins (B, D1, D2, D3, E, F and G) and three U1-specific proteins (U1-70K, U1-A and U1-C), is a critical component of the splicing machinery that regulates both constitutive and alternative splicing (Buratti and Baralle, 2010). U1 snRNP plays an essential role in spliceosome assembly and recognition of the 5′ splice site (5′ss) through both RNA-protein interactions and base-pairing of U1 snRNA with the sequences at the 5′ss defined by the last three nucleotides of the exon and the first six nucleotides of an intron (Du and Rosbash, 2002; Lund and Kjems, 2002; Ptok et al., 2019). Interaction of U1 snRNA with the 5′ss can occur in different registers in the vicinity of the 5′ss with no apparent effect on splicing outcomes (Tan et al., 2016). Growing evidence supports that U1 snRNP can influence the selection of the 5′ss through sequences away from the 5′ss in the absence of the usual recruitment of U1 snRNP at the 5′ss (Singh R. N. and Singh N. N. 2019). Independent of 5′ss selection, U1 snRNP can affect spliceosome assembly by interacting with factors that define the 3′ss (Barabino et al., 1990). Other than spliceosome assembly, U1 snRNP determines the directionality of transcription and transcript length through interactions with both the transcription machinery and the newly synthesized RNA (Venters et al., 2019). There also exists a unique RNP, U1-TAF15 snRNP, encompassing entirely different protein components but containing exactly the same U1 snRNA present in the U1 snRNP (Jobert et al., 2009). U1-TAF15 snRNP and its components have been implicated in transcription and/or splicing regulation (Jobert et al., 2009; Ibrahim et al., 2013). For the sake of simplicity, here we refer to any RNP assembled on U1 snRNA as U1 RNP, including U1 snRNP and U1-TAF15 snRNP. As per the exon definition model of splicing, cross-exon interactions facilitated by recruitment of a single U1 snRNP at the 5′ss play an important role in defining exonic sequences (De Conti et al., 2013). However, limited attention has been paid towards the intron-definition model in which cross-intron interactions could be facilitated by potential recruitment of multiple U1 RNPs at 5′ss-like sequences within intronic sequences. In other words, it is not known if the interaction of U1 RNPs with 5′ss-like sequences within the intron serves as an important initial step towards defining the boundaries of an exon.

Spinal muscular atrophy (SMA) is a leading genetic disease of infants and children (Awano et al., 2014; Ahmad et al., 2016; Wirth et al., 2020). SMA is caused by homozygous deletion of or mutations in the *Survival Motor Neuron 1* (*SMN1*) gene which codes for the essential SMN protein (Singh R. N. et al., 2017). Humans carry a second copy of the *SMN* gene, *SMN2*, which fails to compensate for the loss of *SMN1* due to predominant skipping of exon 7 (Lorson et al., 1999; Monani et al., 1999a). Transcripts lacking exon 7 of *SMN2* produce a truncated protein, SMNΔ7, which is less stable and is only partial functional (Vitte et al., 2007; Burnett et al., 2009; Cho and Dreyfuss, 2010; Gray et al., 2018). Strategies aimed at the restoration of *SMN2* exon 7 inclusion are approved therapies for SMA (Singh et al., 2020). For the sake of simplicity, we use *SMN1*/*2* to refer to both *SMN1* and *SMN2* as these two genes are expressed at the same level and are spliced similarly except for exon 7 (Echaniz-Laguna et al., 1999; Monani et al., 1999b). Multiple factors and cis-elements have been implicated in regulation of *SMN1*/*2* exon 7 splicing (Singh and Singh, 2018). It has been also established that the 5′ss of *SMN2* exon 7 is presented within an inhibitory context encompassing several negative cis-elements, including intronic splicing silencer N1 (ISS-N1), a terminal stem-loop structure and an intronic structure facilitated by a long-distance interaction (Singh et al., 2004b, 2006, 2007, 2013; Hua et al., 2008; Beusch et al., 2017; Frederiksen et al., 2021). Interaction of TIA1, an SMA modifying factor, with sequences immediately downstream of the 5′ss of exon 7 of *SMN2* has been proposed to enhance the recruitment of U1 snRNP and promote exon 7 inclusion (Singh et al., 2011; Howell et al., 2017a). Consistently, engineered U1 snRNAs (eU1s) with extended base pairing at the 5′ss of exon 7 restore *SMN2* exon 7 inclusion (Singh et al., 2007). Also, eU1s targeting intronic sequences located downstream of the 5′ss of exon 7 restore *SMN2* exon 7 inclusion, supporting the role of U1 RNP in splice site selection from a distance (Rogalska et al., 2016; Singh et al., 2017a).

In addition to exon 7, exons 3 and 5 of *SMN1*/*2* undergo skipping alone or in combination with other exons, as captured by a multi-exon skipping detection assay (MESDA) (Singh et al., 2012; Seo et al., 2016b). We previously reported that the inhibition of U1 RNP in HeLa cells using an antisense oligonucleotide (ASO) triggers skipping of multiple internal exons of *SMN1*/*2* (Singh et al., 2017a). However, it is not known if this skipping might be tied to intronic sequences located away from the 5′ss. Recently we reported an *SMN2* super minigene encompassing *SMN2* promoter, all exons and their flanking intronic sequences and the entire 3′ untranslated region (3′UTR) (Ottesen et al., 2024). Despite introns in the super minigene being truncated, *SMN2* super minigene showed a nearly identical splicing pattern to that of the endogenous *SMN2*. Also, inhibition of transcription elongation produced a similar effect on splicing of exons 3 and 7 in the *SMN2* super minigene and endogenous *SMN1*/*2*, suggesting that kinetic coupling of *SMN2* transcription and splicing of exons 3 and 7 is not impacted by intron truncations or the chromatin context specific to the large intronic sequences (Ottesen et al., 2024). At the same time, depletion of DHX9 promoted skipping of exons 3, 4 and 5 in the context of endogenous *SMN1*/*2* but not in the context of the super minigene, underscoring the role of the intronic sequences missing in the context of the super minigene. These results supported that the *SMN2* super minigene could be exploited to examine if the intronic sequences missing from the super minigene have significance under specific situations such as the depletion of splicing factors.

Here we compare the effect of U1 RNP inhibition on removal of each intron of *SMN1*/*2* in HeLa, neuronal SH-SY5Y and SMA patient cells. Our results reveal susceptibility of most internal exons to skipping under conditions of U1 RNP inhibition with the effect being more pronounced on the alternatively spliced exons 3, 5 and 7. We employed the *SMN2* super minigene to determine if deletion of internal sequences within introns would have any bearing on the ability of U1 RNP inhibition to change splicing of *SMN1*/*2* exons. Surprisingly, we observed the loss of the inhibitory effect of U1 RNP inhibition on splicing of exons flanked by the truncated introns. Our findings support a hypothesis that U1 RNPs promote definition of exons in the context of endogenous *SMN1*/*2* through intronic sequences outside the canonical 5′ss present at the exon/intron junction. Supporting this argument, inclusion of none of the internal exons affected by U1 RNP inhibition were fully restored by eU1s targeting their respective 5′ss. Our results reveal a novel post-transcriptional role of U1 RNPs in general splicing regulation through an intron definition model. Our findings are significant for developing novel SMA therapies through elevating U1 RNP levels so that introns of *SMN2* are efficiently excised.

## 2 Materials and methods

### 2.1 Cell culture and transfection

All cell culture reagents and media were obtained from Life Technologies unless otherwise specified. Human cervical adenocarcinoma (HeLa) cells were obtained from the American Type Culture Collection (ATCC) and were grown in Dulbecco’s modified Eagle’s medium (DMEM) supplemented with 10% fetal bovine serum (FBS). SH-SY5Y neuroblastoma cells were obtained from ATCC and grown in a 1:1 mix of minimum essential medium (MEM) and F12 growth medium and supplemented with 10% FBS. GM03813 SMA patient cells were obtained from Coriell Institute of Medical Research and were grown in MEM supplemented with 1X GlutaMAX and 15% FBS. ASOs were obtained from Dharmacon and contained 2′-O-methyl modifications at each base and phosphorothioate backbones. The sequences of oligonucleotides are as follows: 10mer control ASO (non-targeting ASO): UUGCCUUUCU, 11mer anti-U1 ASO: CAGGUAAGUAU. To transfect cells: HeLa and SH-SY5Y cells were counted and seeded at a density of 1 × 10^5^ or 3.5 × 10^5^ cells per well of 24-well plates, respectively. GM03813 cells were counted and seeded at a density of 2.8 × 10^5^ cells per well of 6-well plates. Sixteen hours later, cells were transfected with the indicated plasmids and/or ASOs using Lipofectamine 2000 following the manufacturer’s instructions. All ASO transfections were adjusted to a total concentration of 200 nM using non-targeting 10mer ASO. For co-transfection of the *SMN2* super minigene (*SMN2^Sup^*) and ASO, we used 200 nM of ASO and 0.2 μg of *SMN2^Sup^*. For co-transfections of eU1s and *SMN2^Sup^*, we used 0.25 μg eU1 expression construct or pCI-Neo empty vector and 0.25 μg of *SMN2^Sup^*. For co-transfections of ASO, eU1, and *SMN2^Sup^*, we used 200 nM of ASO, 0.25 μg of eU1 expression construct, and 0.1 μg of *SMN2^Sup^*. For co-transfections using *SMN2∆I6* minigene and eU1s, we used 0.3 μg of *SMN2∆I6* and 0.5 μg of an eU1 expression construct or pCI-Neo empty vector. Six hours after transfection, media containing transfection complexes was replaced with fresh media. Twenty-four hours after transfection, cells were lysed directly in TRIzol reagent (Life Technologies) for RNA isolation.

### 2.2 RNA isolation and multi-exon skipping detection assay (MESDA)

All primers used in this study were previously described (Singh et al., 2004a; Ottesen et al., 2024). RNA was isolated using TRIzol reagent (Life Technologies) following the manufacturer’s instructions. After isolation, RNA was treated with RQ1 RNase-free DNase (Promega) to remove contaminating DNA, following the manufacturer’s instructions. RNA was then re-purified by phenol:chloroform extraction and ethanol precipitation. To generate cDNA, reverse transcription (RT) was carried out in 5 μL reactions containing 0.5–1.0 μg RNA using Superscript III reverse transcriptase (Life Technologies) following the manufacturer’s instructions. To generate cDNA for qPCR and MESDA, RT reactions were primed with random primers (Promega) and gene-specific primers, respectively.

MESDA was carried out as previously described (Singh et al., 2012) using 5′ end-^32^P-labeled reverse primer. For *SMN2^Sup^* MESDA, the forward primer annealed within the FLAG tag region and reverse primer annealed to the first 25 bases of exon 8, while for endogenous MESDA, the forward primer annealed to the exon 1 region across the ATG start codon, which is interrupted by the FLAG tag in *SMN2^Sup^* (Ottesen et al., 2024). Splice isoforms were quantified by densitometric quantification using ImageJ software. For each isoform, percentage inclusion was calculated by dividing the intensity of its band by the total signal in each lane.

### 2.3 Quantitative PCR

Quantitative PCR (qPCR) was carried out using PowerUp SYBR green master mix (Life technologies). Each 20 μL reaction contained 3 μL of 1:40 diluted cDNA (equivalent to 7.5 ng RNA) and 0.6 μM of each primer. Templates for standard curves were generated as previously described (Ottesen et al., 2024). Copies/cell were estimated using the assumption that each HeLa cell contains approximately 30 pg. of RNA. For each experiment, *SMN2^Sup^* and endogenous *SMN1*/*2* transcripts were normalized against HPRT by calculating the relative expression of HPRT in each individual sample relative to the average across a given experiment, then dividing each *SMN2^Sup^* or *SMN1*/*2* measurement by that value.

### 2.4 Sequence analysis

5′ss scores were calculated using MaxEntScan (Yeo and Burge, 2004). Putative intronic 5′ss-like sequences were identified using ESEFinder 3.0 and mapped to the *SMN1/2* locus similar to Cartegni et al. (2003) and Kaida et al. (2010).

### 2.5 Statistical analysis

Excel (Microsoft, Version 16.62) was used for all calculations and generation of plots. Data was expressed as mean ± SEM. *p* values were calculated using an unpaired Student’s t-test. Unless otherwise mentioned, experiments were performed in triplicate, and *p* values were two-tailed and the level of statistical significance was set as *p* < 0.05.

## 3 Results

### 3.1 Effect of inhibition of U1 RNP on transcription and splicing of *SMN1*/*2*

**Figure 1:**
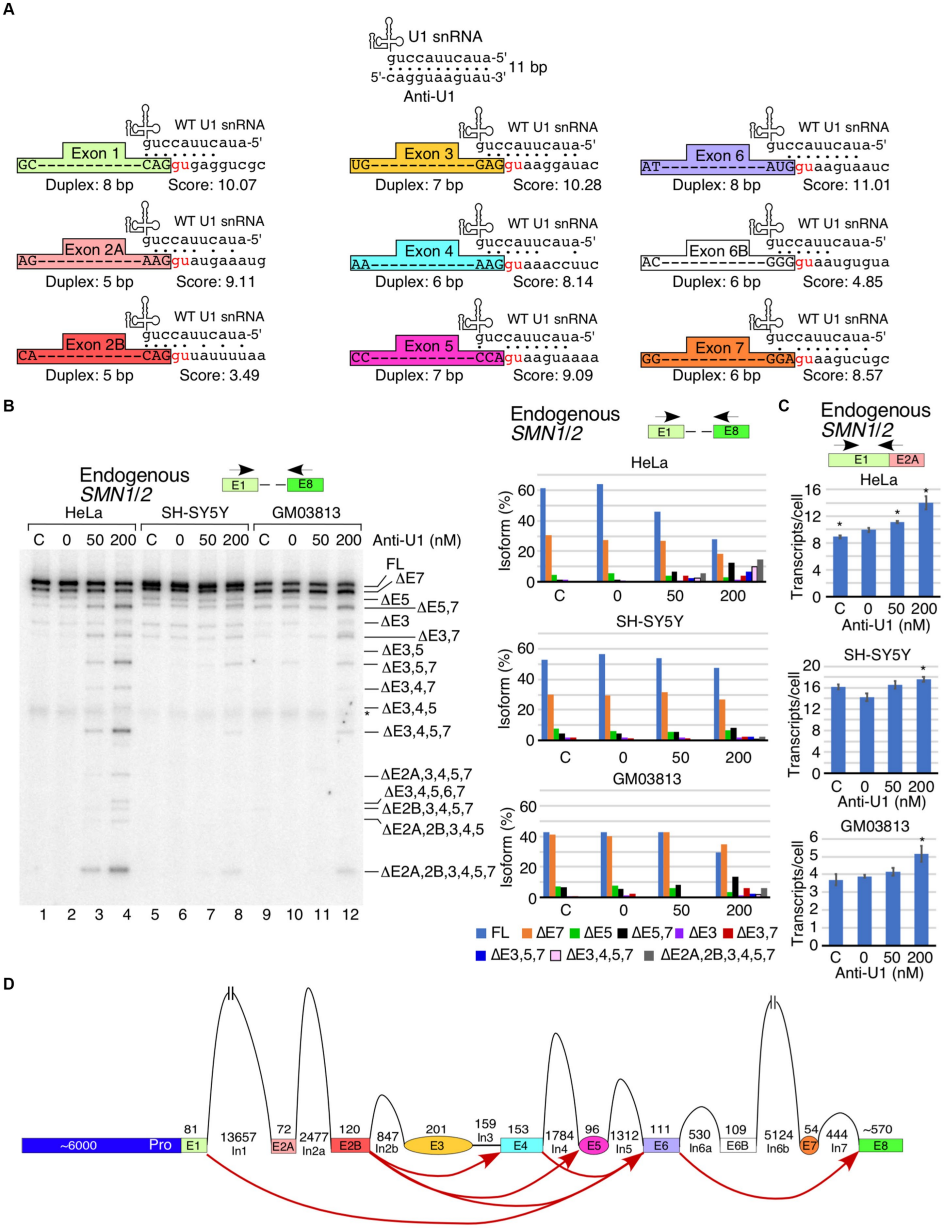
Cell type- and exon-specific effect of U1 RNP inhibition on *SMN1/2* transcripts. **(A)** Diagrammatic overview of WT U1 snRNA and its interactions with anti-U1 ASO or each of the 5′ss of *SMN1/2* exons. Exons are shown as different colored boxes. Dots indicate base pairing between U1 snRNA and anti-U1 or 5′ss. GU dinucleotides that make up the first two intronic positions of each 5′ss are shown in red. The lengths of the longest contiguous stretch of base pairs (bp) in each interaction and splice site scores are indicated. “Anti-U1” indicates anti-U1 ASO. **(B)** Left panel: Splicing pattern of endogenous *SMN1*/*2* transcripts isolated from HeLa, SH-SY5Y, or GM03813 cells transfected with anti-U1 ASO as determined by MESDA. Locations of primers used are indicated above the gel. Cell types and treatments are labeled at the top. “C” indicates untransfected control cells and “0” indicates transfection of nontargeting control ASO. Splice isoforms are labeled at the right side of the gel. “*” indicates a nonspecific background signal. Right panels: Quantification of relative isoform abundance depicted in the left panel. Isoform color coding is indicated at the bottom. Cell type is indicated above each graph. **(C)** Total transcript level of endogenous *SMN1*/*2* in cells transfected with anti-U1 ASO as determined by qPCR. Locations of primers used are indicated at the top. Cell type is indicated above each graph. Error bars represent the standard error of the mean (SEM), *n* = 3. ^*^p < 0.05. **(D)** Diagrammatic overview of the *SMN1*/*2* genes and exon skipping events triggered by anti-U1 ASO. Exons are shown as colored shapes, introns as black lines/broken lines. Red curved arrows denote splicing events triggered by anti-U1 ASO.

In order to determine the effect of inhibition of U1 RNP on transcription and splicing of *SMN1/2*, we transfected cells with 50 or 200 nM of anti-U1 ASO that sequesters the first 11 nucleotides at the 5′ end of U1 snRNA (Figure 1A). As a control, we used a 10 nucleotide-long non-targeting ASO. We performed this experiment in three cell types: HeLa cells, SH-SY5Y neuroblastoma cells, and GM03813 SMA patient fibroblasts. Of note, the 5′ss of an exon forms an RNA:RNA duplex with the U1 snRNA during the early stages of spliceosome assembly. A strong 5′ss:U1 snRNA duplex is expected to be favorable for defining the 5′ss, particularly at low concentrations of functional U1 snRNP. Each exon of *SMN1/2* has a different sequence at the 5′ss that forms a unique 5′ss:U1 snRNA duplex (Figure 1A). Our approach is likely to capture any correlation between the size of the 5′ss:U1 snRNA duplex and exon inclusion under conditions of U1 snRNP inhibition. We employed MESDA to analyze the splicing of all internal exons of *SMN1/2*. In HeLa cells, both concentrations of anti-U1 ASO decreased the proportion of full-length transcript of *SMN1/2*, while skipping of multiple exons was increased, especially at 200 nM (Figure 1B). The strongest effect was observed on the alternatively spliced exons 3, 5 and 7 as we captured increased levels of co-skipped transcripts such as ∆5,7, ∆3,7, and ∆3,5,7. We also captured enhanced skipping of constitutive exons 2A, 2B and 4 in different combinations along with skipping of exons 3, 5 and 7. The most prominent short transcript was ∆2A,2B,3,4,5,7 in which only exons 1, 6, and 8 were included. These results supported that the inhibition of U1 snRNP by an anti-U1 ASO was not absolute and still allowed recognition of strong 5′ss. Of note, exons 1 and 6 happen to have the strongest 5′ss:U1 snRNA duplex (8 bp) among all exons of *SMN1/2*. When we adjusted conditions to capture extremely short transcripts of *SMN1*/*2*, we detected a very faint band corresponding to the skipping of all internal exons of *SMN1*/*2* (Supplementary Figure S1). However, this band was observed only when HeLa cells were transfected with the highest concentration of anti-U1 ASO. As compared to HeLa cells, the effect of anti-U1 ASO on splicing of *SMN1/2* exons was less pronounced in case of SH-SY5Y and GM03813 cells (Figure 1B). We attribute this to the decreased transfectability of neuronal SH-SY5Y and GM03813 cells compared to HeLa cells, although we cannot rule out that these cell types might have relatively high tolerances for decreased levels of functional U1 RNPs. To measure the impact of U1 RNP inhibition on total *SMN1*/*2* mRNA levels, we performed qPCR using primers annealing to exon 1 and the junction between exons 1 and 2A (Figure 1C). Use of these primers allows capture of a vast majority of spliced isoforms of *SMN1/2* as well as any prematurely terminated transcripts that contain at least the first two exons, although variants lacking exon 2A will be missed. Transfection alone caused a slight increase in *SMN1/2* levels in HeLa cells, while, surprisingly, there was a concentration-dependent increase in total *SMN1/2* levels upon transfection with anti-U1 ASO (Figure 1C, top panel). This trend was also captured in case of SH-SY5Y and GM03813 cells, although the effect was significant only at 200 nM of anti-U1 ASO (Figure 1C, lower two panels). Overall, our results support a model in which skipping of multiple exons was caused by pairing of splice sites separated by large distances, such as between the 5′ss of exon 2B and the 3′ss of exon 6 (Figure 1D).

### 3.2 Intronic sequences away from the splice sites are essential to mediate the effect of U1 RNP on splicing of *SMN1*/*2* exons

**Figure 2:**
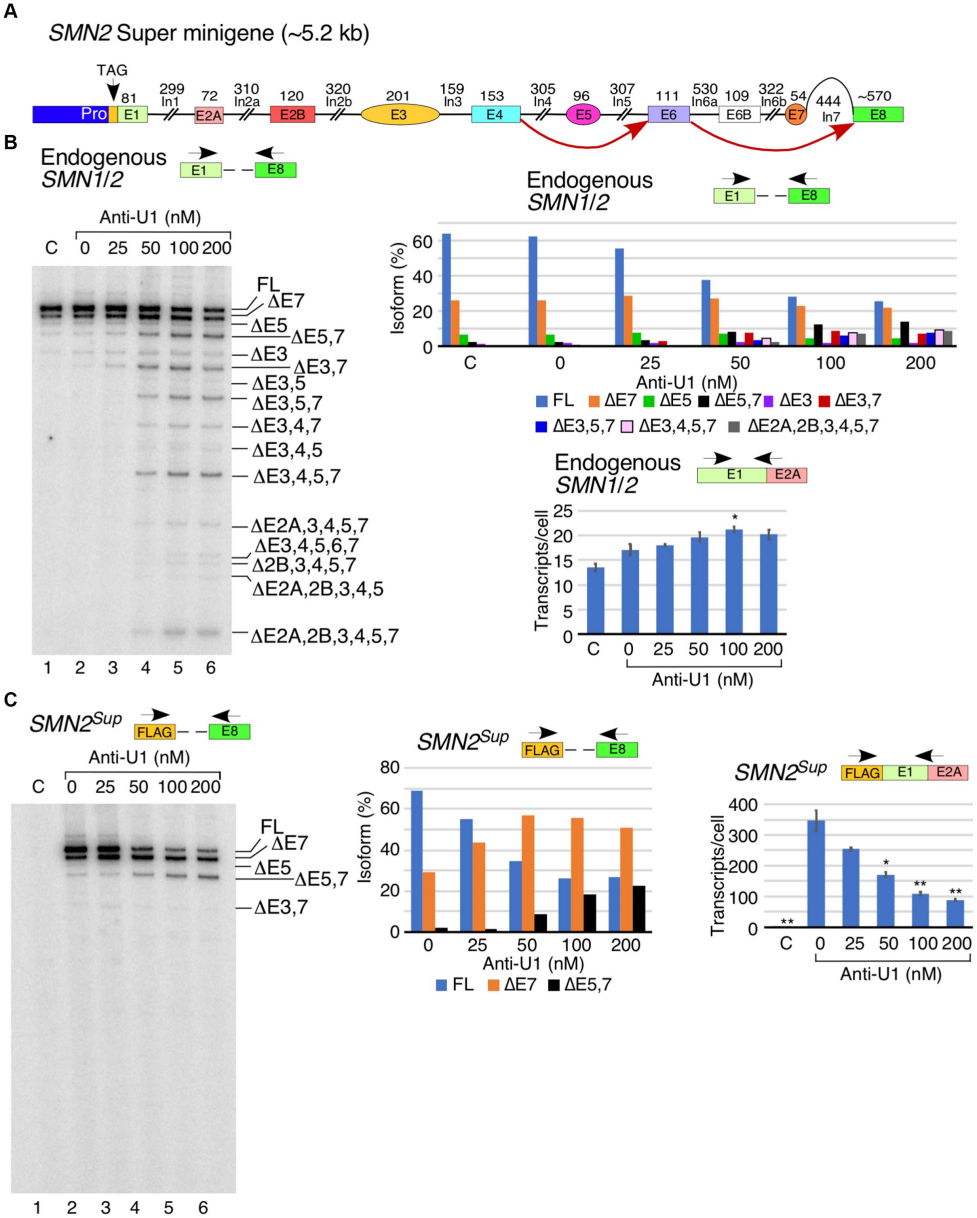
Comparison of the effect of anti-U1 ASO on endogenous *SMN1*/*2* and *SMN2* super minigene. **(A)** Diagrammatic overview of the *SMN2* super minigene (*SMN2^Sup^*) and exon skipping events triggered by anti-U1 ASO. Exons are shown as colored shapes, introns as black lines/broken lines. Red curved arrows denote splicing events triggered by anti-U1 ASO. **(B)** Left panel: Splicing pattern of endogenous *SMN1*/*2* transcripts isolated from HeLa cells co-transfected with anti-U1 ASO and *SMN2^Sup^* as determined by MESDA. Locations of primers used are indicated above the gel. Anti-U1 treatments are labeled at the top. “C” indicates untransfected control cells and “0” indicates transfection of non-targeting control ASO. Splice isoforms are labeled at the right side of the gel. Upper right panel: Quantification of relative isoform abundance depicted in the left panel. Isoform color coding is indicated at the bottom. Lower right panel: Total transcript level of endogenous *SMN1*/*2* transcripts isolated from HeLa cells co-transfected with anti-U1 ASO and *SMN2^Sup^* as determined by qPCR. Locations of primers used are indicated at the top. Error bars represent SEM, *n* = 3. ^*^p < 0.05. **(C)** Left panel: Splicing pattern of *SMN2^Sup^* transcripts isolated from HeLa cells co-transfected with anti-U1 ASO and *SMN2^Sup^* as determined by MESDA. Labeling is the same as in **(B)**. Middle panel: Quantification of relative isoform abundance depicted in the left panel. Isoform color coding is indicated at the bottom. Right panel: Total transcript level of *SMN2^Sup^* transcripts isolated from HeLa cells co-transfected with anti-U1 ASO and *SMN2^Sup^* as determined by qPCR. Locations of primers used are indicated at the top. Error bars represent SEM, *n* = 3. ^*^p < 0.05, ^**^p < 0.01.

We have recently reported an *SMN2* super minigene (*SMN2^Sup^*) encompassing all internal exons flanked by their intronic sequences but carrying large internal deletions within introns (Figure 2A) (Ottesen et al., 2024). In order to examine the potential role of intronic sequences away from splice sites in U1 RNP-dependent splicing of *SMN1*/*2* exons, we transfected HeLa cells with *SMN2^Sup^* and different concentrations of anti-U1 ASO. We monitored splicing using MESDA with primers designed to specifically amplify transcripts generated from either *SMN2^Sup^* or endogenous *SMN1*/*2*. As expected, increasing concentrations of anti-U1 ASO promoted skipping of the alternatively spliced exons 3, 5 and 7 as well as constitutive exons 2A, 2B and 4 in different combinations in case of endogenous *SMN1*/*2* (Figure 2B). Surprisingly, in case of *SMN2^Sup^*, we only observed an increase in skipping of exon 7 and co-skipping of exons 5 and 7. Even the highest concentration of anti-U1 ASO had no effect on splicing of any of the early exons, including the alternatively spliced exon 3, in transcripts generated from *SMN2^Sup^* (Figure 2C). We observed a reduction in intensity of MESDA bands for *SMN2^Sup^* but not for endogenous *SMN1*/*2*, suggesting that the inhibition of U1 RNP had a negative effect on levels of transcripts generated from *SMN2^Sup^* which contains shortened introns. This observation was confirmed by the results of qPCR that showed a clear decrease in *SMN2^Sup^* transcript levels with the increasing concentrations of anti-U1 ASO (Figure 2C, right panel). In contrast, expression of endogenous *SMN1*/*2* moderately increased at all anti-U1 ASO concentrations, although the effect was statistically significant only at 100 nM of anti-U1 ASO (Figure 2B, lower right panel). Notice that earlier described increase in the levels of endogenous *SMN1*/*2* transcripts upon U1 RNP inhibition was observed in HeLa cells that were not simultaneously transfected with *SMN2^Sup^* (Figure 1C). Hence, less impressive rise in the levels of the endogenous *SMN1*/*2* presented in Figure 2B could be attributed to the presence of *SMN2^Sup^* transcripts in the same cells.

### 3.3 Effect of overexpression of engineered U1 snRNAs targeting the 5′ss of *SMN1*/*2* exons

**Figure 3:**
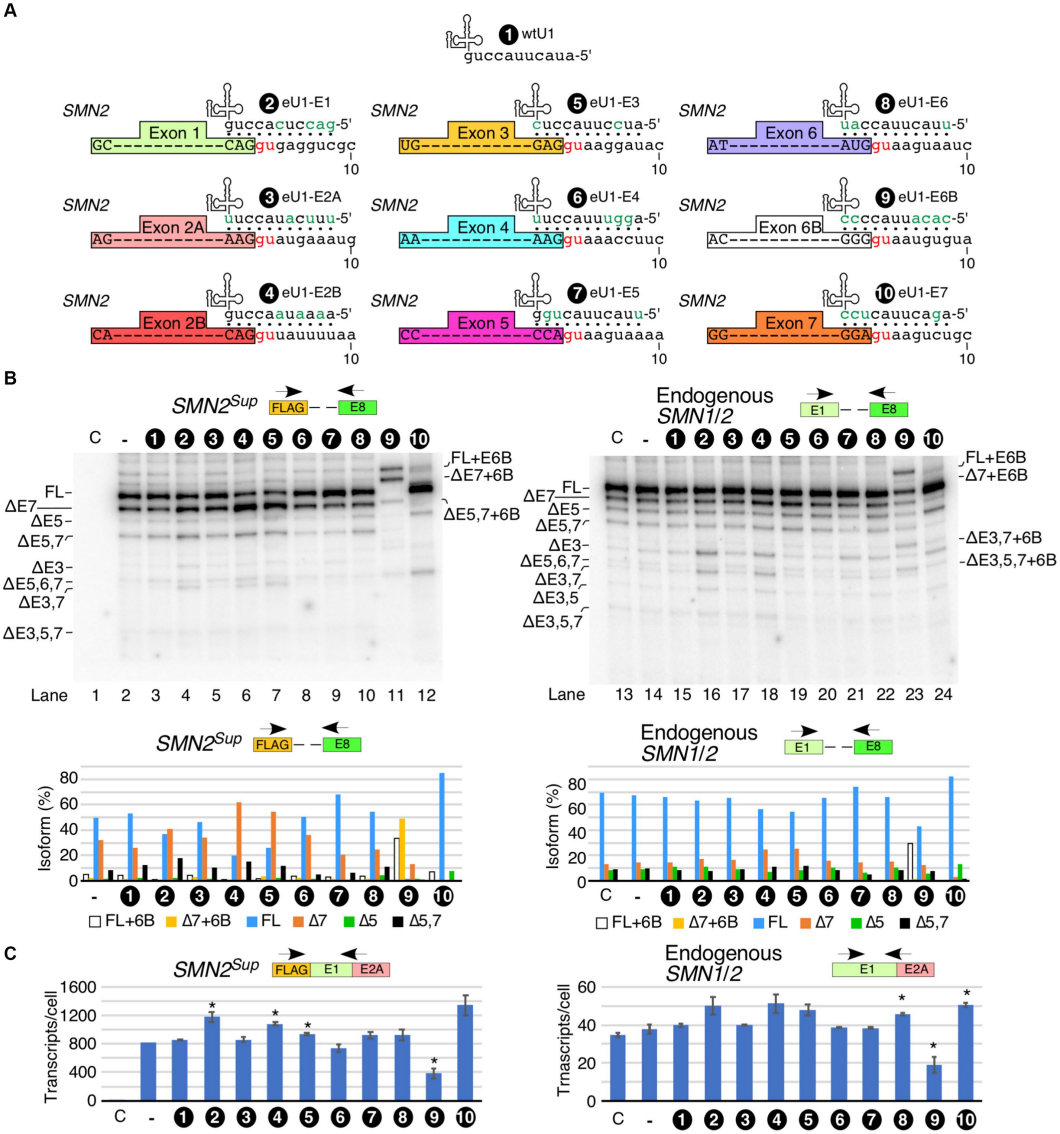
Effect of engineered U1 snRNA overexpression on splicing of *SMN1/2* exons. **(A)** Diagrammatic representation of base pairing formed between eU1s and the 5′ss of *SMN1*/*2* exons. Three nucleotides upstream and eight nucleotides downstream of each GU are shown. The GU dinucleotides are indicated by red letters. Base pairing is marked by black dots; mutated nucleotides within U1 snRNAs are indicated in green. **(B)** Splicing pattern of *SMN2^Sup^* (left panel) and endogenous *SMN1*/*2* (right panel) transcripts isolated from HeLa cells in the presence of the overexpressed eU1s shown in **(A)** as determined by MESDA. The identity of eU1 constructs is marked at the top of the gel. Splice isoforms are indicated at the left and right sides of each gel. “C” indicates untransfected control cells and “-“indicates transfection of *SMN2^Sup^* only. Quantification of the relative amount of the indicated splice products is given in the lower panel as a bar diagram. **(C)** Total transcript level of *SMN2^Sup^* (left panel) and endogenous *SMN1*/*2* (right panel) transcripts isolated from HeLa cells in the presence of eU1 overexpression. Locations of primers used are indicated at the top. Error bars represent SEM, *n* = 3. ^*^p < 0.05 compared to wtU1 control.

Having examined the effect of U1 RNP inhibition on splicing of *SMN1*/*2* exons, we next tested the effect of overexpression of eU1s on splicing of each exon of *SMN1*/*2*. Prior studies support that increasing the size of the 5′ss:U1 snRNA duplex has an stimulatory effect on inclusion of exons (Singh, 2007; Zychlinski et al., 2009; Hicks et al., 2010; Singh et al., 2017a). We designed eU1s such that the size of the 5′ss:U1 snRNA duplex of the target exon is increased to 11 bp (Figure 3A). We transfected HeLa cells with eU1 snRNA-expressing plasmids along with *SMN2^Sup^* and monitored splicing by MESDA (Figure 3B). Compared to control, overexpression of eU1s targeting early exons of *SMN1*/*2*, namely eU1-E1, eU1-E2A, eU1-E2B, and eU1-E3, triggered an increase in skipping of exon 7, and to a lesser extent, co-skipping of exons 5 and 7 in *SMN2^Sup^* transcripts (Figure 3B, left panel). eU1-E1, eU1-E2B, and eU1-E3 also increased co-skipping of exons 3 and 7 in *SMN2^Sup^* transcripts. The fact that an eU1 targeting the 5′ss of exon 3 was somewhat deleterious for its splicing indicates that the nature of the 5′ss of exon 3 does not contribute significantly to its skipping. eU1-E4 and eU1-E6 did not significantly alter splicing of any of the exons of *SMN2^Sup^* transcripts, supporting that the recruitment of U1 snRNP at the 5′ss of the constitutively spliced exons 4 and 6 is not a limiting factor for splicing of the neighboring alternatively spliced exons 3, 5 and 7 (Figure 3B, left panel). Surprisingly, although eU1-E5 did not change splicing of exon 5 by itself, it significantly reduced skipping of exon 7 and co-skipping of exons 5 and 7 in *SMN2^Sup^* transcripts (Figure 3B, left panel). We have previously shown that a cryptic exon (exon 6B) derived from an Alu element in intron 6 is included in some transcripts of *SMN1* and *SMN2* (Seo et al., 2016a; Ottesen et al., 2017). Co-transfection of eU1-E6B with *SMN2^Sup^* resulted in complete inclusion of exon 6B in full-length transcripts as well as exon 7-skipped and exon 5- and 7-co-skipped splice isoforms generated from *SMN2^Sup^*, with no effect on splicing of other exons (Figure 3B, left panel). Overexpression of eU1-E7 triggered almost complete inclusion of exon 7 in *SMN2^Sup^* transcripts as demonstrated by the disappearance of the ∆7 band and drastic increase of the full-length (FL) band (Figure 3B, left panel). Consistently, co-skipping of exons 5 and 7 was decreased while skipping of exon 5 alone was increased. Unexpectedly, there was an increase in co-skipping of exons 5, 6, and 7 in *SMN2^Sup^* transcripts in the presence of overexpressed eU1-E7.

We examined the effects of overexpression of eU1s on splicing of endogenous *SMN1/2* in the same samples transfected with *SMN2^Sup^*. Overall, the effects of eU1s were less prominent on endogenous *SMN1/2* transcripts than on *SMN2^Sup^* transcripts, likely due to the presence of a high background of untransfected cells (Figure 3B, right panel). However, we noted increased skipping of exon 3 and co-skipping of exons 3 and 7 caused by overexpression of eU1-E1 and eU1-E2B, and increased skipping of exon 7 triggered by overexpression of eU1s targeting exons 2B and 3 (Figure 3B, right panel). Overexpression of eU1-6B promoted inclusion of exon 6B in full-length transcripts as well as transcripts with skipped exons 3, 5, and 7 in different combinations (Figure 3B, right panel). To capture the non-specific effect of eU1s on splicing of exon 7, we employed the *SMN2∆I6* minigene that lacks sequences upstream of exon 6 and carries a large internal deletion within intron 6 (Singh et al., 2004a). As expected, overexpression of eU1-E7 almost completely restored inclusion of exon 7 in transcripts generated from *SMN2∆I6* (Supplementary Figure S2). Consistent with the results observed for *SMN2^Sup^*, overexpression of eU1-E1 and eU1-E3 increased skipping of exon 7, whereas eU1-E5 promoted inclusion of exon 7 in transcripts generated from *SMN2∆I6* (Supplementary Figure S2).

We tested how overexpression of eU1s affected levels of transcripts derived from *SMN2^Sup^*. Overexpression of eU1s targeting the 5′ss of exons 1, 2B and 3 showed statistically significant increases in transcript levels (Figure 3C). eU1s targeting the 5′ss of exons 5, 6 and 7 showed increase in transcripts generated from *SMN2^Sup^* as well, although the effects were not statistically significant. In case of eU1 targeting the 5′ss of exons 6B, we observed a noticeable decrease in levels of transcripts generated from *SMN2^Sup^*. This is likely due to susceptibility of the exon 6B-containing transcripts to NMD as previously reported (Seo et al., 2016a). Effects of eU1s on endogenous *SMN1*/*2* transcript levels were largely similar to those we observed for transcripts produced from *SMN2^Sup^*, although the effects were smaller probably due to the high background of untransfected cells (Figure 3C).

### 3.4 Counteracting the effects of U1 RNP inhibition with engineered U1 snRNAs

**Figure 4:**
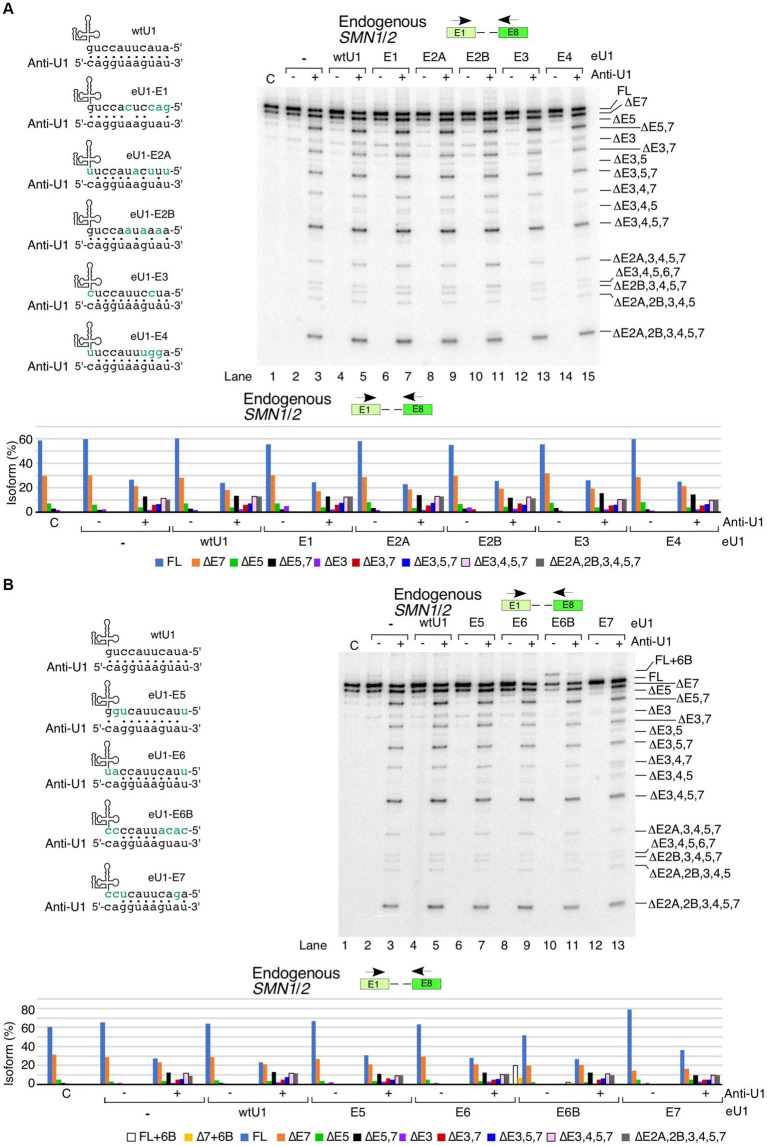
Functional interactions between engineered U1 snRNAs and anti-U1 ASO affecting splicing of endogenous *SMN1*/*2* transcripts. **(A)** Effect of co-transfection of anti-U1 ASO and eU1s targeting early exons of *SMN1*/*2*. Upper left panel: Base pairing between eU1s targeting early exons of *SMN1*/*2* and anti-U1. Coloring and labeling are the same as in Figure 3A. Upper right panel: Splicing pattern of endogenous *SMN1*/*2* isolated from HeLa cells co-transfected with 200 nM anti-U1 or control ASO and plasmids expressing the indicated eU1s, as determined by MESDA. Primer locations are indicated. The target exon of each eU1 and absence (−) or presence (+) of anti-U1 are indicated at the top. “C” – untransfected control. Splice isoforms are labeled at the right side of the gel. Lower panel: Quantification of relative isoform abundance depicted in the top right panel. Isoform color coding is indicated at the bottom. **(B)** Effect of co-transfection of anti-U1 ASO and eU1s targeting late exons of *SMN1*/*2*. All labeling and coloring are the same as in **(A)**.

We asked whether U1 RNP inhibition by anti-U1 ASO could be counteracted by the overexpression of eU1s targeting specific exons. We based our experiment on the premise that the 11-bp-long duplex formed between eU1 and its target 5′ss would be thermodynamically more stable than the 8-bp or shorter duplex formed between anti-U1 ASO and an eU1. Therefore, we hoped to capture *SMN1/2* exons that are vulnerable to skipping due to small size of the duplex formed between the 5′ss and U1 snRNA. Among eU1s that targeted the first five exons, eU1-E3 and eU1-E4 partially neutralized the negative effect of anti-U1 ASO (Figure 4A). These results confirmed that the small size of the duplex formed between the 5′ss and U1 snRNA contributes at least in part towards the skipping of exon 3 and 4 under conditions of inhibition of U1 snRNP. However, our results do not rule out the possibility that the stimulatory effect of eU1s could be due to interaction with the same deep intronic motifs that engage with the wild type U1 RNP. Our results supported that the inhibitory effect of the anti-U1 ASO is not associated with the poor recruitment of the U1 snRNP to the 5′ss of exons 2A and 2B and the effect of inhibition of U1 RNP is likely exerted by intronic sequences away from the splice sites. Of note, considering that the effect of U1 snRNP inhibition on splicing of exons 2A and 2B was not captured in the context of super minigene, we rule out that poor recruitment of eU1s at the 5′ss of exons 2A and 2B is the reason behind their skipping under conditions of inhibition of U1 snRNP (Figure 2). Overexpression of eU1-E3 and eU1-E4 had the most prominent “rescue” effects on ∆3,4,5,7, which is one of the major isoforms produced by the inhibition of U1 RNP (Figure 4A). We also observed a clear reduction in the levels of several other small isoforms, including ∆2A,3,4,5,7, ∆3,4,5,6,7, ∆2B,3,4,5,7, ∆2A,2B,3,4,5, and ∆2A,2B,3,4,5,7 upon overexpression of eU1-E3 and eU1-E4 in the presence of anti-U1 ASO (Figure 4A). Overexpression of eU1-E5 was unable to counteract the inhibitory effect of anti-U1 ASO on splicing of exon 5 of *SMN1*/*2*, suggesting that the recruitment of U1 snRNP at the 5′ss of exons 5 is not the limiting factor for splicing and the effect of inhibition of U1 RNP is likely exerted by intronic sequences away from the splice sites (Figure 4B). Splicing of exon 6 is not impacted by the inhibition of U1 RNP. Consistently, overexpression of eU1-E6 had no effect on splicing of exon 6 with or without anti-U1 ASO (Figure 4B). Surprisingly, a strong RNA:RNA duplex formed between the 5′ss of exon 6B and eU1-6B lost most of its ability to enhance exon 6B inclusion in the presence of anti-U1 ASO. These results indicate that the small RNA:RNA duplex formed between the 5′ss and U1 snRNA is one of the major limiting factors for the inclusion of the exon 6B in full-length transcripts of *SMN1*/*2*. We observed a partial rescue of exon 7 inclusion by eU1-E7 in the presence of anti-U1 ASO (Figure 4B).

**Figure 5:**
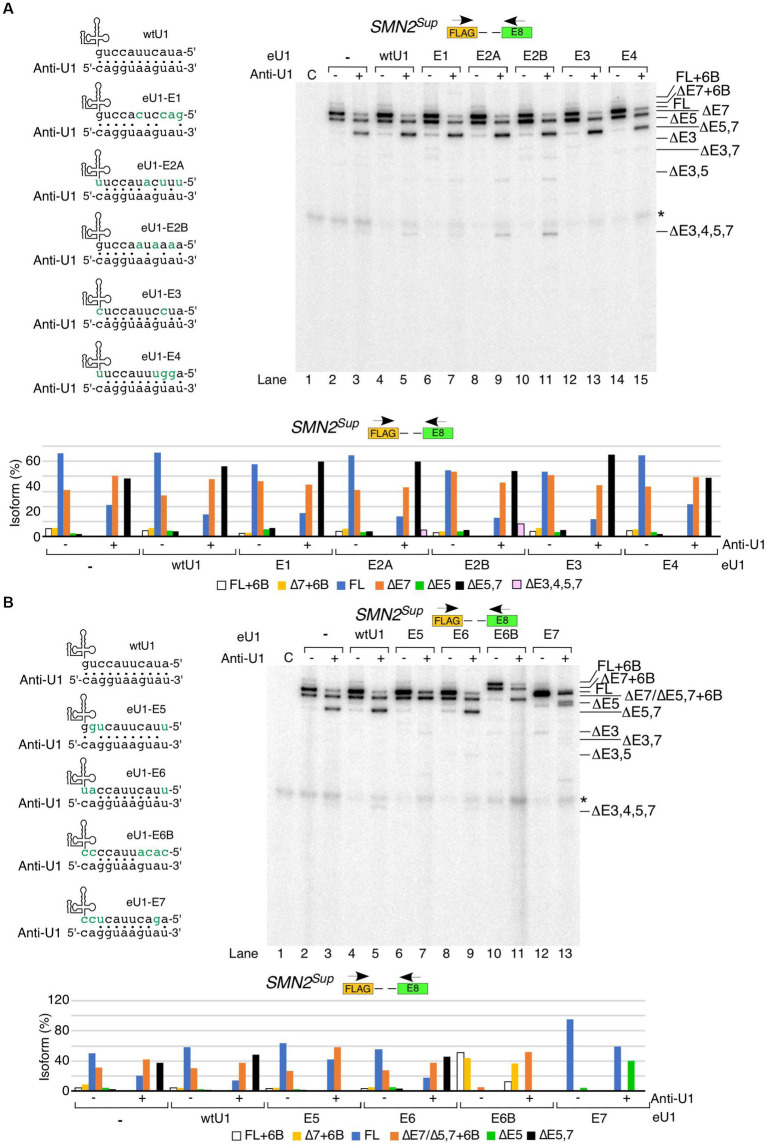
Functional interactions between engineered U1 snRNAs and anti-U1 ASO affecting splicing of *SMN2^Sup^* transcripts. **(A)** Effect of co-transfection of anti-U1 ASO and eU1s targeting early exons of *SMN2^Sup^*. Upper left panel: Base pairing between eU1s targeting early exons of *SMN1*/*2* and anti-U1. Coloring and labeling are the same as in Figure 3A. Upper right panel: Splicing pattern of *SMN2^Sup^* transcripts isolated from HeLa cells co-transfected with 200 nM anti-U1 or control ASO, plasmids expressing the indicated eU1s, and *SMN2^Sup^*, as determined by MESDA. Primer locations are indicated. The target exon of each eU1 and absence (−) or presence (+) of anti-U1 are indicated at the top. “C” – untransfected control. Splice isoforms are labeled at the right side of the gel. “*” indicates a nonspecific background signal. Lower panel: Quantification of relative isoform abundance depicted in the top left panel. Isoform color coding is indicated at the bottom. **(B)** Effect of co-transfection of anti-U1 ASO and eU1s targeting late exons of *SMN2^Sup^*. All labeling and coloring are the same as in **(A)**.

We performed similar rescue experiments in the context of *SMN2^Sup^* by co-transfecting it with anti-U1 ASO and eU1s (Figure 5). As in case of the endogenous *SMN1*/*2* transcripts, eU1s targeting early exons did not prevent the negative effects of anti-U1 ASO on splicing of exons 5 and 7 in *SMN2^Sup^* transcripts (Figure 5A). In fact, eU1-E3 had a noticeable additive inhibitory effect on splicing of exons 5 and 7 in the presence of anti-U1 ASO. Interestingly, eU1-E2A and eU1-E2B triggered enhanced skipping of exons 3 and 5 in the presence of anti-U1 ASO, as shown by a slight increase in the levels of ∆3,5 and ∆3,4,5,7 isoforms (Figure 5A). Unlike the effect of eU1-E5 on splicing of the endogenous *SMN1*/*2* transcripts, this eU1 was able to fully counteract the inhibitory effect of anti-U1 ASO on splicing of exon 5 in transcripts derived from *SMN2^Sup^* transcripts (Figure 5B). In addition, eU1-E5 reduced the impact of anti-U1 ASO on skipping of exon 7. Likewise, eU1-E6B retained its ability to promote exon 6B inclusion in the context of *SMN2^Sup^* transcripts even in the presence of anti-U1 ASO, although it was not able to prevent skipping of exon 7 or co-skipping of exons 5 and 7 (Figure 5B). Of note, due to their similar sizes, exon 7-skipped transcripts and transcripts in which exons 5 and 7 are co-skipped along with exon 6B inclusion (∆5,7 + 6B) are indistinguishable by electrophoresis. Therefore, we confirmed the identity of the product in question by cloning and sequencing (Figure 5B, lane 11). eU1-E7 completely prevented skipping of exon 7 in *SMN2^Sup^* transcripts in the presence of anti-U1 ASO, but did not have a strong positive impact on splicing of exon 5 (Figure 5B). However, considering we and others have previously shown that the recruitment of eU1s at cryptic 5′ss downstream of the canonical 5′ss of *SMN2* exon 7 promote inclusion of exon 7, we cannot rule out that the stimulatory effect of eU1-E7 on exon 7 splicing is not due to the interaction of eU1-E7 with intronic motifs downstream of the 5′ss of exon 7.

## 4 Discussion

Here we report a novel role of U1 RNP in transcription and splicing regulation through intronic sequences located away from the 5′ss. Unlike previous studies that captured the effect of strong U1 RNP inhibition using a 25-mer ASO on transcription and splicing during the process of transcription elongation (Kaida et al., 2010; Berg et al., 2012), we have focused on the effect of U1 RNP inhibition on splicing events that likely occur post-transcriptionally. We used a shorter 11-mer ASO to specifically block the critical 5′-end of U1 snRNA while not perturbing the assembly of the U1 snRNP. Use of shorter ASO was also aimed at reducing the off-target effects. Of note, longer ASOs are known to produce more off-target effects than shorter ASOs (Scharner et al., 2020; Ottesen et al., 2021). Our study was enabled by the employment of MESDA that specifically captured the relative abundance of all spliced transcripts containing the first and the last annotated exons. Our approach eliminated the complex analysis of short transcripts generated upon U1 snRNP inhibition due to random pre-mature cleavage and polyadenylation. Inhibition of U1 snRNP led to the enhanced skipping of almost all exons in different combinations. We observed similar effects in all cell types examined, although the effect was more pronounced in HeLa cells (Figure 1). This is likely due to relatively high sensitivity of HeLa cells to U1 RNP inhibition and/or an efficient transfection of ASO used for the U1 snRNP inhibition. Notably, inhibition of U1 RNP caused a slight increase in the levels of endogenous *SMN1*/*2* transcripts as measured by qPCR using primers that annealed to early exons (Figure 1C). Inhibition of U1 RNP is associated with premature transcription termination (Di et al., 2019). Therefore, it is likely that U1 RNP inhibition leads to an increase in levels of the only truncated transcripts generated from endogenous *SMN1*/*2*. Suppression of transcription has been linked to increased association of U1-TAF15 snRNP with RNA polymerase II (Jobert et al., 2009). Hence, it is likely that anti-U1 ASO decreases the association of U1-TAF15 snRNP with RNA polymerase II, leading to the enhanced transcription of *SMN1*/*2* we captured in this study.

**Figure 6:**
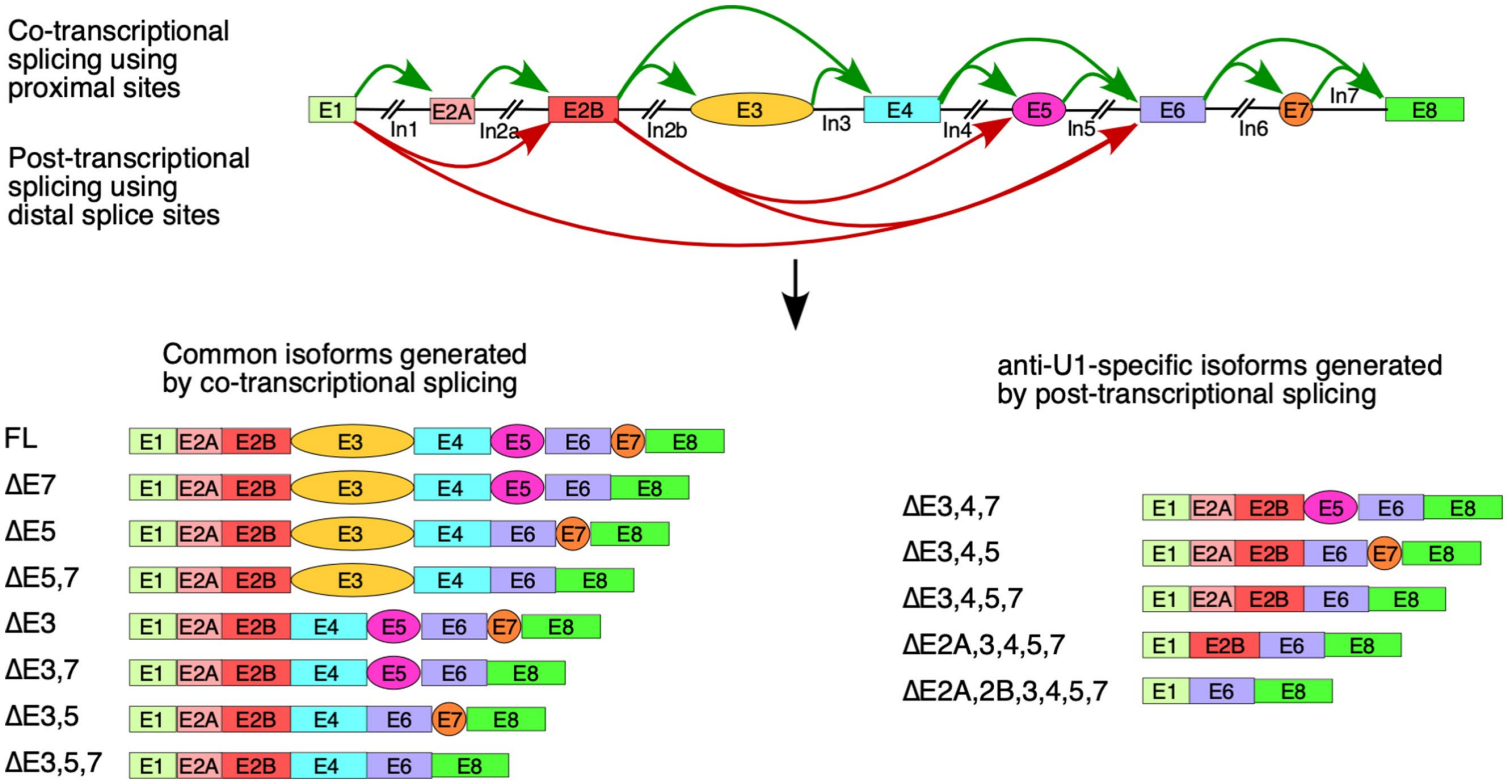
Model of co-transcriptional and delayed splicing of *SMN1*/*2* exons. Diagrammatic overview of the *SMN1*/*2* genes. Exons are shown as colored boxes and introns as black lines/broken lines. Green curved arrows indicate splice site pairings between nearby exons supported by co-transcriptional splicing model. Red curved arrows indicate splice site pairings using distal splice sites that are triggered by anti-U1 ASO. Graphical representations of major splicing products are shown below.

Based on the profile of endogenous *SMN1*/*2* transcripts affected by U1 RNP inhibition, skipping of the alternative exons 3, 5 and 7 appeared to have occurred largely independently of each other (Figure 1B). Consistently, we detected different combinations of co-skipping that involved exons 3, 5, and 7. We detected skipping of exon 4 along with skipping of exons 3 and 5, suggesting that the co-skipping of exons 3, 4 and 5 is a single event enabled by pairing of the 5′ss of exon 2B with the 3′ss of exon 6 (Figures 1D, 6). We also observed a prominent transcript lacking the first five internal exons. We attribute generation of this transcript to pairing of the 5′ss of exon 1 with the 3′ss of exon 6 (Figures 1D, 6). It appears that the co-skipping of exons 2A, 2B, 3, 4 and 5 competes with co-skipping of exons 3, 4 and 5 since the 3′ss of exon 6 is used for both of these events. We observed very small but detectable levels of transcripts lacking all internal exons, suggesting that pairing of the 5′ss of exon 1 with the 3′ss of exon 8 of *SMN1*/*2* pre-mRNA is a rare event with or without U1 RNP inhibition (Supplementary Figure S1). The most abundant short transcript produced upon U1 RNP inhibition harbored exon 6, suggesting that the 3′ss and/or 5′ss of exon 6 are the strongest splice sites among all internal exons of *SMN1*/*2*. Consistently, the 5′ss of exon 6 forms the longest (8 bp) RNA:RNA duplex with U1 snRNA as compared to other 5′ss of internal exons of *SMN1*/*2* (Figure 1A). Considering inclusion of exon 6 was not affected under the conditions of inhibition of U1 snRNP supports that the concentration of the residual functional U1 snRNP was sufficient to promote exon 6 inclusion through the relatively strong RNA:RNA duplex formed between U1 snRNA and the 5′ss of exon 6. Of note, U1 snRNP is not absolutely required for splicing of certain exons (Fukumura et al., 2009). Hence, it remains a possibility that the splicing of exon 6 of *SMN1*/*2* belongs to the category of exons that are not regulated by U1 snRNP.

U1 snRNP interacts directly with the elongating polymerase II and can remain tethered throughout transcription of the downstream introns (Leader et al., 2021; Zhang et al., 2021). This allows for efficient linear splicing as the proximal 3′ss to a given 5′ss is brought in very close proximity during transcription, allowing for immediate formation of the spliceosomal complexes. Anti-U1 ASO appears to prevent or interfere with such co-transcriptional splicing, particularly that of introns 1 and 2B, allowing for co-skipping of exons 2A through 5 or exons 3 through 5 (Figure 6). This may be due to a delayed interaction of U1 snRNP with its target 5′ss, preventing it from forming a functional complex with elongating polymerase II, or it may be due to disruption of the U1 snRNP-polymerase II complex during transcription of long introns, discouraging co-transcriptional splicing. Future studies will show whether treatment with anti-U1 ASO affects the levels of *SMN1*/*2* circular RNAs (Ottesen et al., 2019), which also require interactions between introns separated by multiple exons.

Employment of the *SMN2* super minigene allowed evaluation of the effect of U1 RNP inhibition on splicing of all internal exons in the context of large deletions in the middle of introns. Surprisingly, U1 RNP inhibition did not impact the splicing of any of the first five exons (Figure 2C), supporting that the limited supply of U1 RNP does not influence the definition of these early exons when flanked by short introns. However, splicing of the alternatively spliced exon 5 that was also flanked by the relatively short introns was adversely impacted by U1 RNP inhibition. Splicing of the alternatively spliced exon 7 that was flanked by the relatively large introns was adversely impacted as well. Our findings may support a novel role of U1 RNP in splicing regulation through an intron definition model, in which U1 RNP is recruited at multiple sites within introns allowing cross-intron communication that favors pairing of the 5′ss with the adjacent 3′ss. Consistently, all of the introns of *SMN1/2* except for the relatively short introns 2B, 3 and 7 contain at least one putative GU-containing-5′ss-like sequence that may serve as a binding site for U1 RNP (Supplementary Figure S3). Interaction of U1 snRNP with intronic sequences that may not resemble putative GU-containing-5′ss but still capable of forming RNA:RNA duplex is possible (Parada et al., 2014; Erkelenz et al., 2018). However, distribution of such sequences is not accurately predictable. Intronic sequences of *SMN1*/*2* harbor inverted Alu repeats capable of forming inhibitory secondary structures that are unwound by DHX9, an RNA helicase (Ottesen et al., 2019; Singh N. N. and Singh N. R. 2019). Consistently, depletion of DHX9 promotes skipping of internal exons in the context of the endogenous *SMN1*/*2* transcripts, but not in transcripts derived from the *SMN2* super minigene carrying large intronic deletions and, hence, lacks Alu elements (Ottesen et al., 2024). Therefore, it is likely that the recruitment of U1 RNPs within intronic sequences abrogates secondary structures formed by the inverted Alu repeats.

Interactions of U1 RNP with chromatin structure impacts all steps of transcription, including initiation, elongation and termination (Chiu et al., 2018; Fiszbein et al., 2019; Zhang et al., 2021; Mimoso and Adelman, 2023). Chromatin is also formed on plasmid DNA, although its nature is dictated by the plasmid size (Shao et al., 2019). Unlike in the case of the endogenous *SMN1*/*2* transcripts, inhibition of U1 RNP significantly decreased the levels of the *SMN2* transcripts generated from the super minigene (Figure 2C). This could be due to adverse effects on one or more steps associated with transcription, including initiation, elongation and termination. Of note, our super minigene contains a short promoter encompassing only the most highly utilized transcription start sites. It is possible that these transcription start sites are differentially susceptible to inhibition of U1 RNPs. It is also possible that inhibition of U1 RNP leads to an increase in the rate of premature termination of transcription during initiation and/or promoter proximal pause (Kamieniarz-Gdula and Proudfoot, 2019). Future studies employing gene-editing based approaches would reveal any correlation between the transcription start site and rate of transcription termination during promoter proximal pause of *SMN1/2*. Such studies may also uncover the effect of the 5′UTR on selection of the first exon and vice versa as recently reported (Fiszbein et al., 2019). We have recently shown that inhibition of transcription elongation triggers skipping of exon 3 in the context of both the super minigene and endogenous *SMN1*/*2* (Ottesen et al., 2024). Considering we did not observe skipping of exon 3 in the context of *SMN2^Sup^*-derived transcripts under the conditions of U1 RNP inhibition, we rule out the role of transcription elongation inhibition as a potential mechanism behind reduced levels of *SMN2* transcripts generated from the super minigene. Poor transcription initiation and/or premature termination of transcription appears to be the cause of reduction in the levels of transcripts derived from *SMN2^Sup^* under the conditions of U1 RNP inhibition.

For more than two decades, researchers have focused on *SMN2* exon 7 inclusion as one of the primary avenues for the treatment of SMA (Singh et al., 2017b, 2020). New ideas of combined therapies with multiple compounds affecting splicing and transcription of *SMN2* are being advanced to improve the efficacy of existing therapies (Oh et al., 2020; Marasco et al., 2022; Ottesen et al., 2023; Ottesen and Singh, 2024). Our finding that U1 RNPs could affect splicing outcomes through intronic sequences located away from the splice sites opens up yet additional avenues for SMA therapy. Considering the SMN complex is involved in snRNP biogenesis, even a slight increase in SMN production would elevate the levels of U1 RNP, which in turn would further increase the levels of SMN (Jodelka et al., 2010). Of note, the SMN complex is also proposed to be involved in disassembly of snRNPs and assembly of U1-TAF15 snRNP (Chari et al., 2008). However, the role of U1-TAF15 snRNP biogenesis in SMA and/or other pathological conditions has not yet been investigated. An inhibitory sequence located in the middle of intron 7 is known to modulate *SMN2* exon 7 splicing (Singh et al., 2010, 2013; Howell et al., 2017b). Future studies will reveal how U1 RNP-responsive deep intronic sequences within *SMN2* could be exploited to enhance SMN levels. To a broader significance, our findings uncover a novel role of U1 RNP in splicing regulation that is independent of spliceosome assembly and intimately tied to deep intronic sequences. Mutations of U1 snRNA and/or misregulation of U1 RNP is associated with many pathological conditions, including amyotrophic lateral sclerosis, Alzheimer’s disease, and cancer (Yu et al., 2015; Bai, 2018; Shuai et al., 2019; Suzuki et al., 2019; Oh et al., 2020). Our findings may provide a mechanistic basis as to how low levels of U1 RNPs may impact splicing of specific genes due to the presence of their responsive cis-elements within deep intronic sequences. Many variants of U1 snRNAs are expressed in humans (O'Reilly et al., 2013). Future experiments will determine if some of the effects of inhibition of U1 snRNP is due to inhibition of the less expressed variants of U1 snRNAs. Overexpression of U1 snRNP is also associated with pathological conditions (Cheng et al., 2017, 2018; Kumari et al., 2019). It would be of interest to see if intronic sequences away from the 5′ss engage in aberrant splicing under conditions of the overexpression of U1 snRNP. An intronic mutation within the *ATM* gene leading to the abrogation of a putative U1 snRNA binding site away from the 5′ss of the upstream exon is associated with ataxia-telangiectasia (Pagani et al., 2002). Therefore, we envision many scenarios in which deep intronic mutations associated with the interaction with U1 snRNP could lead to pathological conditions.

## Data availability statement

The original contributions presented in the study are included in the article/Supplementary material, further inquiries can be directed to the corresponding author.

## Author contributions

EO: Conceptualization, Formal analysis, Investigation, Validation, Writing – original draft, Writing – review & editing. NS: Writing – original draft, Writing – review & editing, Formal analysis, Investigation, Validation. JS: Investigation, Writing – original draft, Writing – review & editing. RS: Conceptualization, Funding acquisition, Supervision, Writing – original draft, Writing – review & editing, Project administration, Resources.
