# Supplementary material for: U1 snRNA interactions with deep intronic sequences regulate splicing of multiple exons of spinal muscular atrophy genes
Source: Front Neurosci. 2024 Jul 12;18:1412893. doi: 10.3389/fnins.2024.1412893 (PMC11289892; doi:10.3389/fnins.2024.1412893)
Supplement: Supplementary file 1 [file Data_Sheet_1.PDF]

U1 snRNA interactions with deep intronic sequences regulate splicing of multiple exons of spinal muscular atrophy genes

Eric W. Ottesen, Natalia N. Singh, Joonbae Seo, Ravindra N. Singh
Department of Biomedical Sciences, Iowa State University, Ames, IA, 50011

SUPPLEMENTARY FIGURES

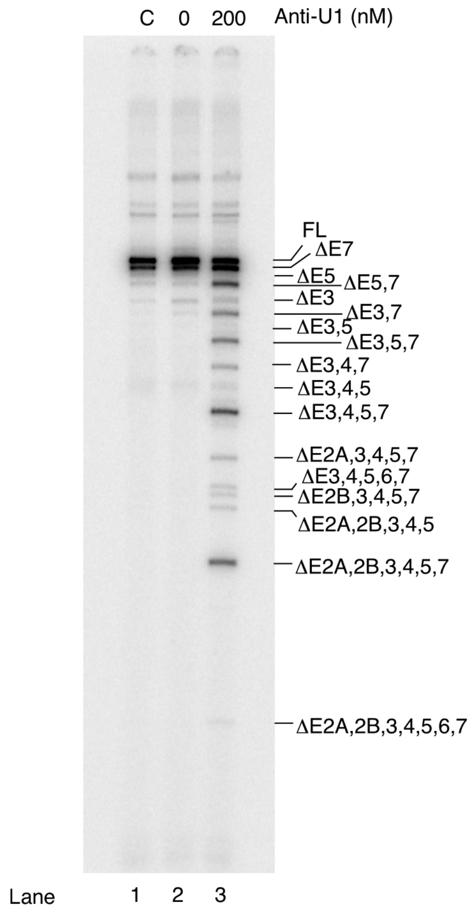

Supplementary Figure 1. Uncropped image of MESDA with limited run time. Alternative splicing of endogenous *SMN1/2* transcripts isolated from HeLa cells transfected with anti-U1 ASO as determined by MESDA. Treatments are indicated at the top. "C" indicates untransfected control cells and "0" indicates transfection of non-targeting control ASO. Splice isoforms are labeled at the right side of the gel.

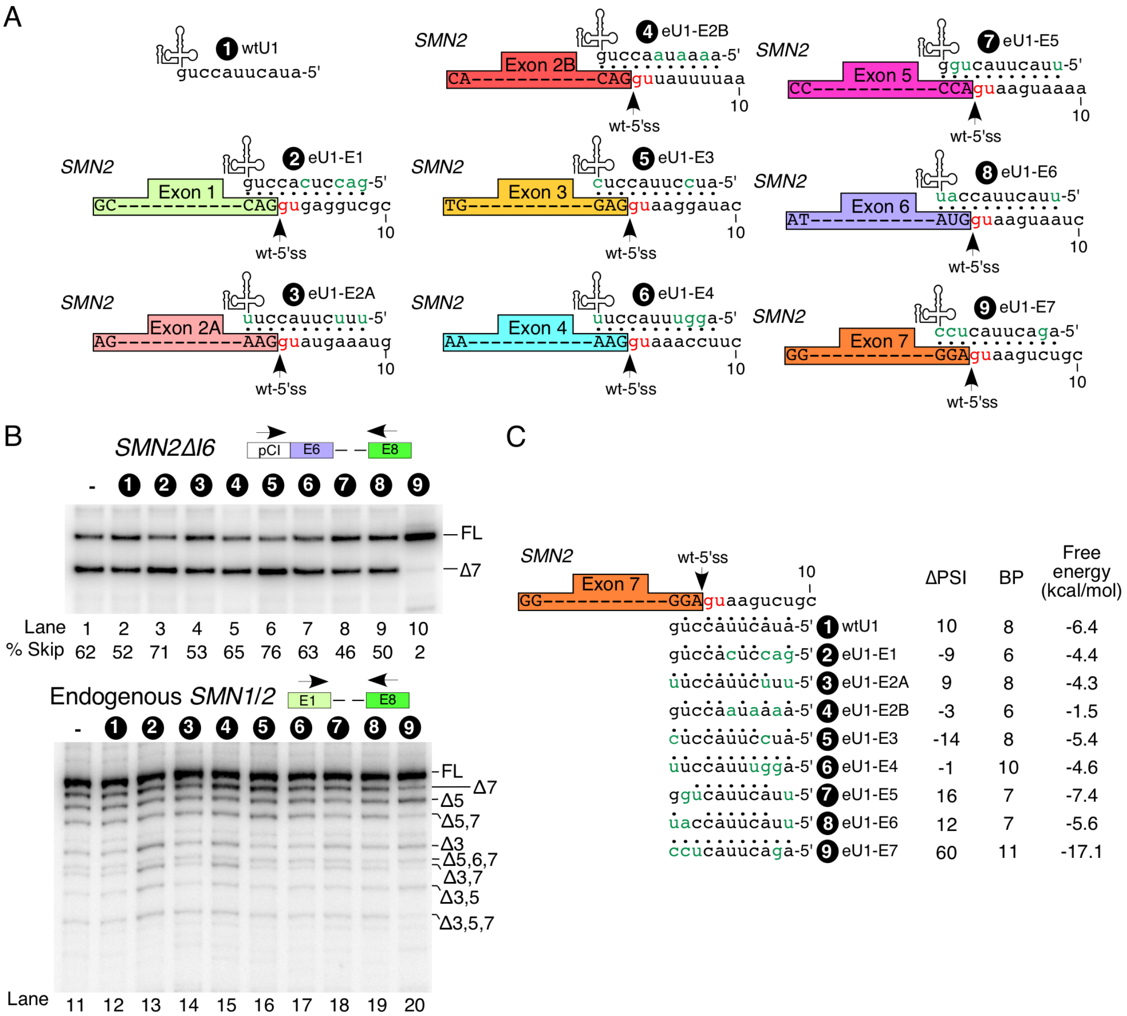

Supplementary Figure S2. eU1s targeting various exons impact *SMN2* exon 7 splicing without the presence of target exons. (A) Diagrammatic representation of base pairing formed between eU1s and exon-5′ss. Coloring and labeling are the same as in Figure 3 **(B)** Semi-quantitative PCR examining splicing of *SMN2ΔI6* (left panels) and MESDA examining splicing of endogenous *SMN1/2* (right panels) in transcripts isolated from HeLa cells co-transfected with plasmids expressing *SMN2ΔI6* and the indicated eU1s. The identity of eU1 constructs is marked at the top of the gel. Splice isoforms are indicated at the left and right sides of each gel. Quantification of splice isoforms are shown below each gel. **(C)** Base pairing between the 5′ss of exon 7 and different eU1s. Coloring is the same as in Figure 3. ΔPSI indicates the change in *SMN2ΔI6* exon 7 splicing. BP indicates total base pairs formed between eU1 and exon 7 5′ss. Free energy indicates the total free energy of the interaction as determined by RNAStructure web server (https://rna.urmc.rochester.edu/RNAstructure.html).

A

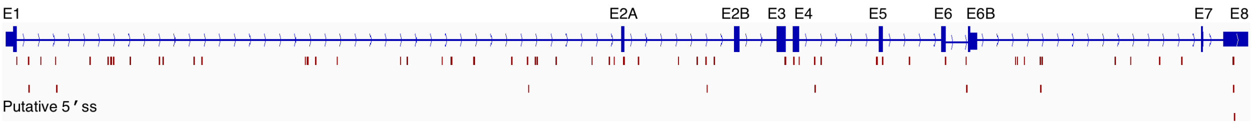

B

Intron 1:

Gene position	Genomic position	Sequence	Score
334	70925438	ACUCCUCUCUGGCUGGUGUGGGGGAGGUGG	6.96
345	70925449	GCUGGUGUGGGGGAGGUGGGGGCAUUAGGC	8.58
614	70925718	CUUGGGCAUAGCAUGGUAGGGGUGAAUUUG	7.29
943	70926047	CGGCCGGAUCACAAGGUCAGGAGAUCGAGA	9.04
975	70926079	AUCCUGGCUAACACGGUGAAACCCCGUCUC	6.84
1720	70926824	AAUGUCCUAGAGUAGGUAAGAAGGGAUGGA	9.77
2131	70927235	CGUGGAUAUGAAGAAGUGAGGGGGAGAAGU	6.73
2201	70927305	UGGCUAAGAAAGGAAGUGAGAGGCCGGGCG	8.12
2258	70927362	CUUUGGGAGACUGAGGUGGGUGGAUUACCU	8.70
2628	70927732	UGGGCAGAUCACAAGGUCAGGAGUUCGAGA	8.18
3292	70928396	UGCAGAGCCUAGAAGGUGAUGGUUAAGAGA	6.99
3376	70928480	AUAAAUACAGUUGAAGUGAGUGAGUAGCUG	7.31
4071	70929175	CUUUUUGUUGCCCAGGUGGGAGUGCAGUGG	8.22
4258	70929362	AAUCUGAAAGUGCAAGUAAGGAGCAGGGAA	8.62
6591	70931695	CCCUGCAAACACCUGGUAUGGUCAGUCUUU	8.38
6638	70931742	GUAAAAAAAGACUAGGUAAGCCGAUAGUUA	9.66
6820	70931924	CUUCACAUACGUAAGGUGGGUAUGUGAAGU	8.91
7304	70932408	AGCCUCACACCCUUGGUGUGUCAGCGUCCU	7.19
8738	70933842	AACUCCUGACCUCAGGUGAUCCACCCACCU	7.79
8889	70933993	ACCCUGGAAAGGCUAGUGAGUACGGUAUUC	7.91
9681	70934785	UAUCUGGGACUACAGGUGUGCACCACCAUG	8.71
9889	70934993	AGACAGAGAUUAUAGGUGUGAGCCACUGGA	7.30
10398	70935502	CUUUGGGAGGCUGAGGUGGGAGGAUCACUU	7.61
11256	70936360	AACUCCUGACCUCAGGUGAUCUGCCCACCU	7.75
11608	70936712	ACUUCCUGGGCCCAGGUGAUCCUCUCGCCU	8.74
11635	70936739	CCUCAGCCCCUGCAGGUAGCUGGGACUAUA	8.16
11785	70936889	GCCGCCCGGGUUCAAGUGAUUCUCCUGCCU	7.59
11827	70936931	CAGCUGGGAUUACAGGUGACUGCCACCACG	9.94
12253	70937357	GCCUCCCAGGUUCAAGUGAUUCUCCUGCCU	6.98
13067	70938171	ACUCAGUGGGCUGAGGUGGGAGGGUCAUUU	8.18
13456	70938560	ACAAAUCUACUCAUGGUAUGUGGAUAGGAA	7.32
13564	70938668	AUAACCUCUAACCAGGUAAGUUUCCUGUGG	13.67

Intron 2A:

Gene position	Genomic position	Sequence	Score
14116	70939220	AGCCUGGCCAACAUGGUGAAACCCCGUCUU	7.46
15020	70940124	UAGUUGGGACCACAGGUGUGUGCCACCACA	10.13
15445	70940549	GAGGUUGAGUUUGAGGUAUGAAUUAAGAUG	7.09
15637	70940741	CGAGCAAAUCACGAGGUCAGGAGUUCAAGA	7.55
15669	70940773	AGCCUGGCCCACAUGGUGAAACCCCGUCUU	7.55
15835	70940939	CAGCCCGGUGACAGAGUGAGACUCUGUGUA	7.11

Intron 4:

Gene position	Genomic position	Sequence	Score
18091	70943195	CUUUGGGAGGCCAAGGUGGGCGGAUCACAA	8.48
18107	70943211	UGGGCGGAUCACAAGGUCAGGAGAUCGAGA	8.18
18237	70943341	UGAGGCAGGAGGAUGGUGUGAACCCCGGAG	6.94
19501	70944605	AUCAUAUUGAAAUUGGUAAGUUAAUUUUCC	7.55

Intron 5:

Gene position	Genomic position	Sequence	Score
20239	70945343	AACUCCUGACCUCAGGUGAUCCACCCGCCU	8.20

Intron 6:

Gene position	Genomic position	Sequence	Score
21520	70946624	GCCUCAGCCUCAGAGGUAGCUGGGACUACA	7.34
21536	70946640	UAGCUGGGACUACAGGUGCCUGCCACCACG	6.85
22630	70947734	GGGAGGUAGAGGCAGGUGGAUCACUUGAGG	6.94
22676	70947780	AGCCUGGCCAACAUGGUGACUACUCCAUUU	7.89
22838	70947942	AGCCUGGCCAACAGAGUGAGAAUUUGCGGA	6.93
23196	70948300	GGGAGGCCAGGGCAGGUGGAUCACGAGGUC	8.04
23208	70948312	CAGGUGGAUCACGAGGUCAGGAGAUCGAGA	7.70
23240	70948344	AUCCUGGCUAACAUGGUGAAACCCCGUCUC	6.71
24885	70949989	GAGGUGGAGGUUGCGGUGAGCCGAGAUCAC	8.01
25234	70950338	AAGGCGGAGGUUGCGGUGAGCCAAGAUUGC	8.37
25884	70950988	CAAAUCCGACCUCAGGUGAUCCGCCCGCCU	8.75
26391	70951495	AACUCCUGAGCUCAGGUGAUCCAACUGUCU	7.29
27556	70952660	CUGUAAAAGACUGAGGUGGGGGUGGGAGGC	8.57
27562	70952666	AAGACUGAGGUGGGGGUGGGAGGCCAGCAC	6.78
27581	70952685	GAGGCCAGCACGGUGGUGAGGCAGUUGAGA	9.09
28160	70953264	UCGUGCCUCACCCACGUGAGUAGUUGGGAU	8.47
28645	70953749	CAAAUCCUACAUUAGGUAAAUGGUUUUGAA	7.33
29287	70954391	AACUCCUGACCCCAGGUAAUCCACCUGCCU	9.47
29674	70954778	GAGGUGGAGGUUGUGGUGAGCCGAGAUUGU	7.91

Supplementary Figure S3. Locations and sequences of intronic 5′ss-like sequences. A) Genomic overview of the *SMN1/2* genes. Exons are shown as blue boxes and introns as lines with arrows indicating the direction of transcription. The locations of all putative 5′ss identified by ESEFinder 3.0 are indicated with red boxes below the *SMN1/2* gene. **B)** Precise locations and sequences of all intronic 5′ss-like sequences. Gene position is given relative to the ATG start codon. Genomic position is given for the *SMN1* locus in the hg38 human genome annotation. The GU dinucleotide for each 5′ss is shown in red and the putative annealing position of U1 snRNA is boxed in grey. Splice site scores were calculated by ESEFinder 3.0.
